# High thermal durability of Ru-based synthetic antiferromagnet by interfacial engineering with Re insertion

**DOI:** 10.1038/s41598-021-94640-4

**Published:** 2021-07-26

**Authors:** Chun-Liang Yang, Chih-Huang Lai

**Affiliations:** Department of Materials Science and Engineering, National Tsing Hua University, No.101, Section 2, Kuang-Fu Road, Hsinchu, 30013 Taiwan

**Keywords:** Magnetic properties and materials, Surfaces, interfaces and thin films

## Abstract

Synthetic antiferromagnets (SAFs), composed of Ru spacer with a Re insertion layer, reveal superior thermal stability up to 450 °C annealing, making the back-end of line process a wider manufacturing window and tolerance to integrate the perpendicular magnetic tunneling junctions (P-MTJs) into CMOS process. The coupling strength decays significantly for SAFs with single Ru spacer after annealing above 400 °C. Due to the characteristics of refractory metals, Re can behave as a diffusion barrier during annealing. Furthermore, the Re spacer can still keep reasonable RKKY coupling strength. Therefore, the SAFs with Ru/Re composite spacers exhibit higher RKKY coupling strength than Ru spacers after 450 °C annealing. In addition, we discovered the different enhancements for the upper and lower interfacial Re insertion, which was attributed to the varied defect formation at interfaces. The stacking fault was formed at the upper Ru/Co interface in as-deposited state. When Re was inserted at the upper interface, the diffusion between Co and Ru was significantly suppressed and the stacking fault can be eliminated during annealing, leading to enhanced interlayer coupling. Through the interfacial engineering, we may have more degrees of freedom to tune the SAF performance and thus enhance process compatibility of P-MTJ to the CMOS process.

## Introduction

Synthetic antiferromagnets (SAF) have played an indispensable role in magnetic tunnel junctions (MTJs) for the applications of magnetic random access memory (MRAM). For high density MRAM, MTJs are typically composed of CoFeB layers with perpendicular magnetic anisotropy^1^. To eliminate the stray field on the free layer, which is generated from the reference layer and may lead to asymmetric switching current density, synthetic antiferromagnetic layer (SAF) with nearly zero magnetization is used as the reference layer^2,3^. SAF is composed of two magnetic layers separated by a spacer with compensation of net magnetic moment through the Ruderman–Kittel–Kasuya–Yosida (RKKY) interaction^4–7^. In addition, the interlayer coupling may enhance stability of the reference layer and prevent it from writing error.

Nowadays, Ru is the most widely utilized material for RKKY spacer in MTJs with large interlayer coupling strength and a suitable crystal structure for perpendicular [Co/Pt]_n_ multilayers as the reference layer^8^. Typically, the Ru thickness is in the range of 0.35–0.45 nm and 0.8–1.0 nm, locating at the 1st and 2nd peaks of RKKY oscillation, respectively, with the coupling strength around 2.0 and 1.0 erg/cm^2^^9,10^. The strongest coupling can be achieved by using the Ru thickness at the 1st peak, however, the thickness control is in a stringent window for fabrication. In addition, to integrate MTJ into CMOS process, an annealing step upon 400 °C or even higher temperature in the back end of line (BEOL) process is required, which may degrade the magnetic properties of MTJ. Since the RKKY coupling, the most important indicator for SAF performance, strongly depends on the interface conditions, this high-temperature annealing may cause significant reduction of the interlayer coupling strength J_ex_ due to the inter-diffusion at the interface between ferromagnetic and Ru layers^10–12^. Recently, Ir has been reported as an alternative spacer, which reveals stronger coupling strength and better thermal durability than Ru. The Ir spacer with the thickness 0.48 nm shows J_ex_ of 1.9 erg/cm^2^ after 400 °C annealing^13^. However, the price of Ir is much higher than that of Ru in material markets. Therefore, searching for the practical and affordable solution of spacer is a key challenge for MRAM mass production.

To prevent diffusion during the high temperature annealing, building a diffusion barrier is an intuitive and effective method. One potential approach is to engineer the interface between Ru and ferromagnetic layers by inserting a thin diffusion barrier. To select a thermally stable material, melting point is a good indicator. Although Mo, Ta and W are of high melting point materials, as known as “refractory metals”, their negligible RKKY coupling and body-centered cubic (BCC) structure are not suitable for the conventional reference layer [Co/Pt] /Ru with face-centered cubic (FCC) and hexagonal close-packed (HCP) structures. On the other hand, another member of refractory metals, Re has a quite high melting point with a HCP structure. Furthermore, Re possesses the forth strongest RKKY coupling strength, while the top three are Ru, Ir, and Rh^14^. Although the coupling strength of Re is not as strong as the top three, it is not negligible like other refractory metals. In this work, we demonstrate a composite spacer composed of Ru and Re, in which Re can reach high thermal durability and still remain RKKY anti-ferromagnetic coupling after annealing upon 450 °C. By optimizing the layer structure, the composite spacer exhibits even higher coupling strength than Ru spacer after 450 °C annealing. Furthermore, since Ru is an essential material for various applications, its large demand in material market leads to a much higher price than Re. Therefore, by engineering Ru interface, we may not only enhance thermal durability of SAFs but manufacture the MTJs in a price-friendly way.

In addition, we also discovered the different enhancements for the upper and lower interfacial Re insertion, which was attributed to the varied defect formation at interfaces during annealing. By the microstructural analyses, we demonstrate that the insertion of Re at the upper interface can eliminate the stacking faults during the annealing, leading to enhanced interlayer coupling. Our findings provide a clear guideline to tune the SAF performance through the interfacial engineering, and can further enhance process compatibility of perpendicular MTJ to the CMOS process.

## Results and discussions

### Structure design

**Figure 1:**
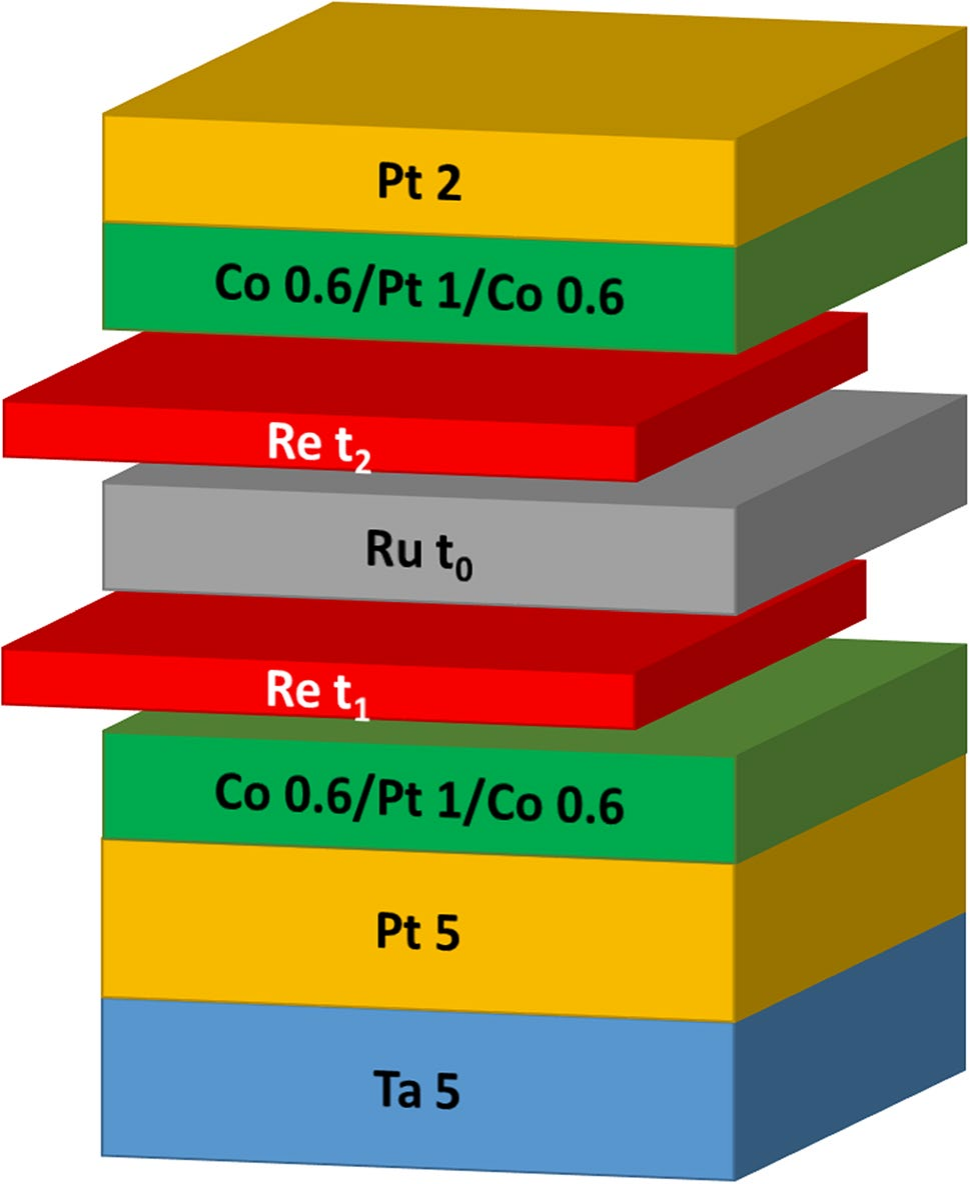
Schematic diagram for film stack of the SAF layer. Re is inserted at top and/or bottom of Ru. The numbers represent layer thickness in nanometers.

We prepared SAF samples with the structure shown in Fig. 1. Based on the typical SAF structure [Co/Pt]_n_/Ru/[Co/Pt]_n_ with perpendicular anisotropy, we additionally insert thin Re film at the interfaces between Ru and Co. As a diffusion barrier, the Re film is expected to perform high thermal durability to prevent interlayer diffusion. The samples were then annealed with varied temperatures up to 450 °C for 1 h, compatible with the thermal budget of BEOL used in CMOS process.

### Magnetic properties of SAFs

We first prepared the samples with a sandwich structure, Re/Ru/Re, in which two Re layers were fixed at 0.18 nm and Ru was varied. The Re layer is about one monolayer inserted between Ru and Co. The sample with a single Ru spacer is also made for comparison. The hysteresis loops were measured by using VSM and the interlayer coupling strength J_ex_ was determined by the following formula^9,15,16^

$$J_{ex} = M_{s} tH_{ex}$$

M_s_ is saturation magnetization of the ferromagnetic layer and t is thickness of ferromagnetic layers. H_ex_, the exchange field strength induced by antiferromagnetic coupling, is the loop shift along the x-axis (field-axis) of M-H loop. The J_ex_ value is plotted as a function of total spacer thickness in Fig. S1 of Supplementary Information. Compared to the sample with a single Ru spacer, the sample with composite spacer reveals similar dependence of interlayer coupling on the spacer thickness, which has a maximum J_ex_ value around 0.79 nm (Ru 2nd RKKY anti-parallel coupling peak). This result indicates that the fixed spacer thickness at 0.79 nm for the composite spacer may still provide the highest coupling strength. Since RKKY strength strongly depends on the spacer thickness, we believe that it is proper to fix the composite spacer at the same thickness for fair comparison with a single Ru spacer. Therefore, for our experimental design, we made all the samples composed of various composite spacers with the same total thickness (0.79 nm). On the other hand, the J_ex_ generated by the Re/Ru/Re spacer was smaller than that by a single Ru spacer because Re provides less coupling strength. Therefore, the bilayer structure with only one side Re is a possible way to reach a balance between the J_ex_ value and thermal durability.

**Figure 2:**
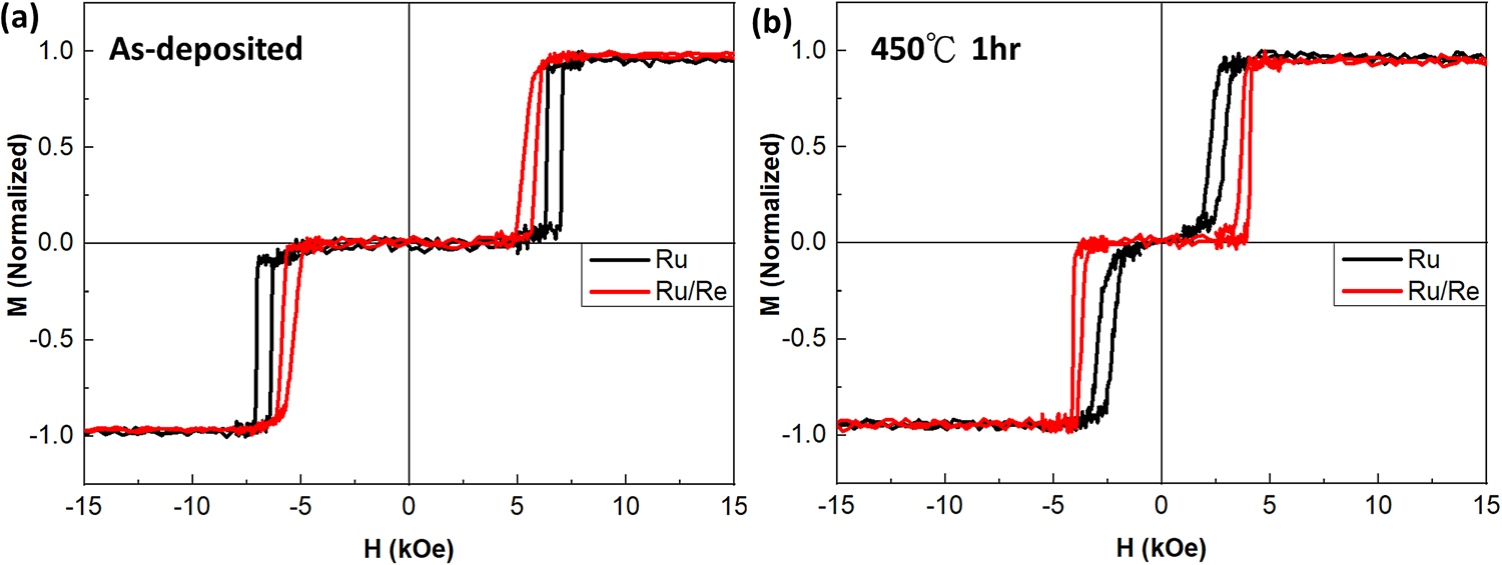
M-H loops of [Co/Pt]_n_ based SAFs. Black curve is for the sample with a single Ru spacer of 0.79 nm and red one is for the sample with a composite spacer of Ru 0.61 nm/Re 0.18 nm. (**a**) As-deposited state (**b**) After 450 °C 1 h annealing.

The sample with a composite spacer, Ru 0.61 nm/Re 0.18 nm, was made to compare with one consisting of a single Ru (0.79 nm) spacer and their hysteresis loops before and after 450 °C annealing are shown in Fig. 2. In the as-deposited state, the largest H_ex_ 6.5 kOe for the sample with a single Ru spacer can be achieved, shown in Fig. 2a, which corresponds to the J_ex_ of 1.02 erg/cm^2^, comparable to the reported value at 2nd RKKY peak^10^. The interlayer coupling J_ex_ is slightly decreased to 0.93 erg/cm^2^ for the as-deposited sample with the Ru/Re composite spacer, shown in Fig. 2a. After annealing at 450 °C for 1 h, J_ex_ degrades to 0.35 erg/cm^2^ for the sample with a single Ru spacer (Fig. 2b), but the J_ex_ value of the sample with a composite spacer is only reduced to 0.57 erg/cm^2^. With the less reduction on J_ex_, the composite spacer shows more robust thermal durability and keeps relatively good J_ex_ after 450 °C annealing.

### Effects of Re insertion on RKKY coupling strength J_ex_

**Figure 3:**
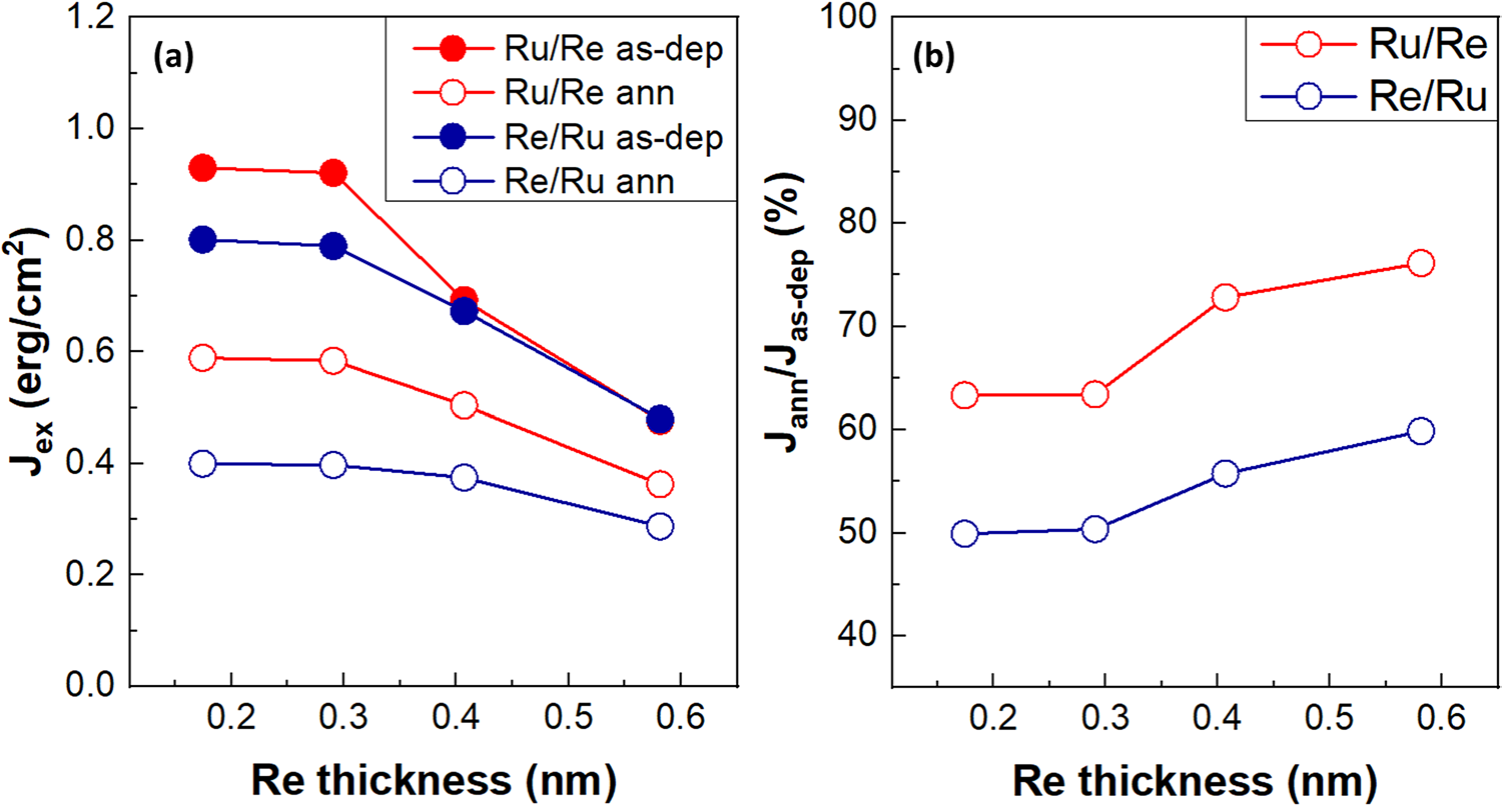
Effects of Re insertion on RKKY interlayer coupling. (**a**) Variations of J_ex_ with Re thickness. The total thickness of the spacer is fixed at 0.79 nm. Red and blue lines represent the Re upper (Ru/Re) and lower (Re/Ru) insertion, respectively. Solid and open circles represent for as-deposited and annealed states, respectively. (**b**) J_ann_/J_as-dep_ presents J_ex_ changes after annealing of Ru/Re (red) and Re/Ru(blue) spacer.

To further explore effects of Re insertion, we prepared the SAF samples with various composite spacers. We made two types of insertion, Ru/Re and Re/Ru, that is, insertion of Re in the upper or lower interface, respectively. In addition, we varied the Ru thickness from 0.18 to 0.61 nm but kept the total spacer thickness about the same (0.79 nm), locating at the region of 2nd AF coupling peak of single Ru. Figure 3a displays the dependence of J_ex_ on Re thickness for the as-deposited and annealed samples. In both spacers, J_ex_ has highest value at Re 0.18 nm (Ru 0.61 nm) and remains similar strength at Re 0.29 nm (Ru 0.50 nm). J_ex_ decreases with further increasing Re thickness, which may result from the smaller RKKY coupling strength of Re. On the other hand, the upper Re insertion (Ru/Re spacer) exhibits higher J_ex_ than the lower Re insertion (Re/Ru spacer). Since J_ex_ strength strongly depends on the Ru (0002) texture grown on (111)-oriented [Co/Pt]_n_ multilayers^17^, we speculate that the thin Re insertion on the top of [Co/Pt]_n_ (the lower insertion case, Re/Ru) may slightly deteriorate the subsequently deposited Ru crystallinity, resulting in a lower J_ex_. After 450 °C annealing, J_ex_ of all samples drops obviously. To verify the crystallinity of Ru and Re, we prepared samples of Si/SiO_2_//Ta 5/Pt 2/Co 0.6/Re (or Ru) 5 nm for XRD measurements, as shown in Fig. S2 of Supplementary Information. The sample with Re shows quite weak signal unlike strong textured Ru, which is consistent with the previous report that Re was not easy to be crystallized when the film thickness is too thin^18^. Therefore, we suggest that very thin Re in our layer structure may grow in relatively poor crystallization on the [Co/Pt]_n_, which slightly deteriorates the following Ru texture. On the other hand, if Ru grows first, the (0002) texture is well established so the following Re can also have (0002) textured growth due to the proper Ru seed layer. Therefore, the different interfacial conditions may lead to various Ru crystallinity, which gives rise to different J_ex_ for composite spacers of Ru/Re and Re/Ru.

To look into details of the changes after annealing, we plot the ratio of J_ex_ changed after annealing, J_ex_ (annealed)/J_ex_ (as-deposited), shown in Fig. 3b, which may indicate the degree of degradation due to inter-diffusion. A higher ratio was observed for thicker Re, suggesting the diffusion is less for thicker Re spacer. In addition, the sample with the upper insertion of Re (Ru/Re spacer) shows better performance, implying that the inter-diffusion may be more severe at the upper interface of spacer so that the suppression of inter-diffusion by upper Re works more effectively.

**Figure 4:**
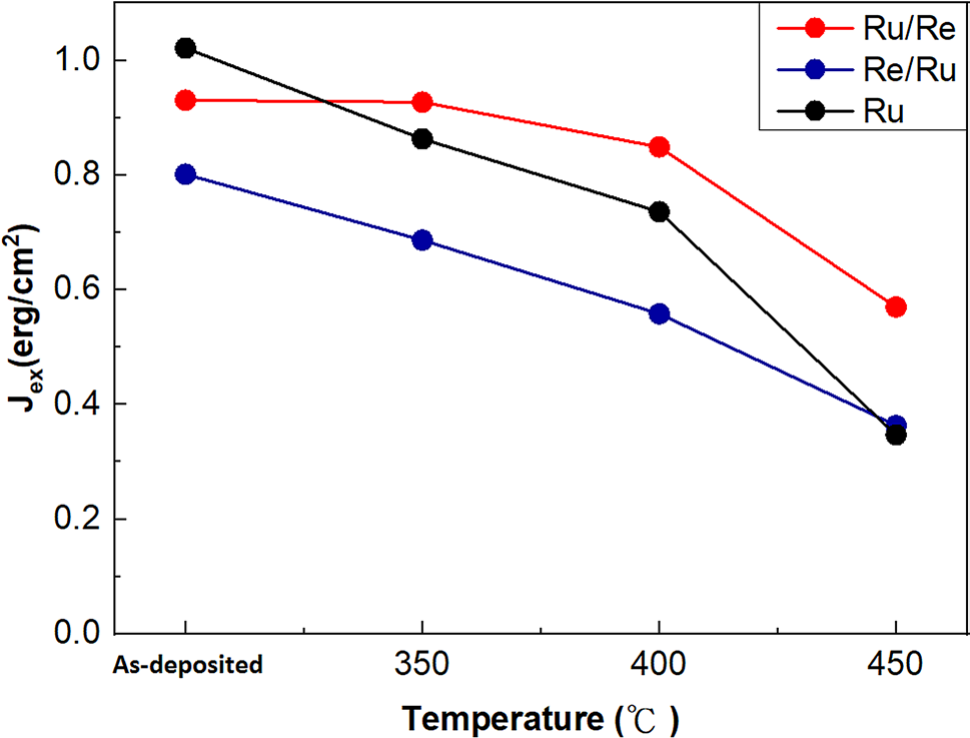
Variations of J_ex_ with different annealing temperature. Red and blue lines are the J_ex_ for the sample with the Ru/Re and Re/Ru spacers, respectively. The thickness for Ru and Re is 0.61 and 0.18 nm, respectively. Black line is the J_ex_ for the sample with Ru spacer (0.79 nm).

We then compare the annealing temperature dependence of J_ex_ for different spacers, as shown in Fig. 4. Compared to the single Ru spacer, the upper insertion (Ru/Re spacer) shows better thermal durability. Although the as-deposited sample with the Ru/Re spacer possesses a slightly lower J_ex_, J_ex_ of the sample with Ru/Re spacer drops slowly and remains a higher value than that of the sample with a single Ru spacer after annealing. In contrast, the thermal durability of lower insertion (Re/Ru spacer) is not as good as Ru/Re. The degradation rate of J_ex_ for the sample with the Re/Ru spacer is similar to that with the Ru spacer up to 400 °C annealing. In addition, the J_ex_ of Re/Ru in the as-deposited state is much lower so that the J_ex_ value of annealed samples is less than that with the Ru spacer for the annealing temperature lower than 450 °C. As we discussed earlier, the insertion of thin Re at the lower interface might deteriorate the Ru crystallinity, leading to the reduced J_ex_.

We also prepared the sandwich spacer composed of Re 0.18 nm/Ru 0.43 nm/Re 0.18 nm. The J_ex_ value is 0.65 erg/cm^2^ in the as-deposited state and becomes 0.40 erg/cm^2^ after 450 °C annealing, slightly higher than the Ru spacer (0.35 erg/cm^2^) but lower than that of the Ru/Re spacer (0.57 erg/cm^2^). Although the insertion of both Re layers may further suppress the inter-diffusion, the deteriorated Ru crystallinity due to the lower insertion and reduced Ru thickness may compensate the gained thermal durability. Consequently, the SAF with the Ru/Re spacer, which can have strong RKKY interaction provided by Ru directly contacting to the ferromagnetic layer at the lower interface and build a barrier to slow down the inter-diffusion at the upper interface, can achieve the highest J_ex_ after high temperature annealing.

### Effects of annealing on interfacial roughness and microstructure

**Figure 5:**
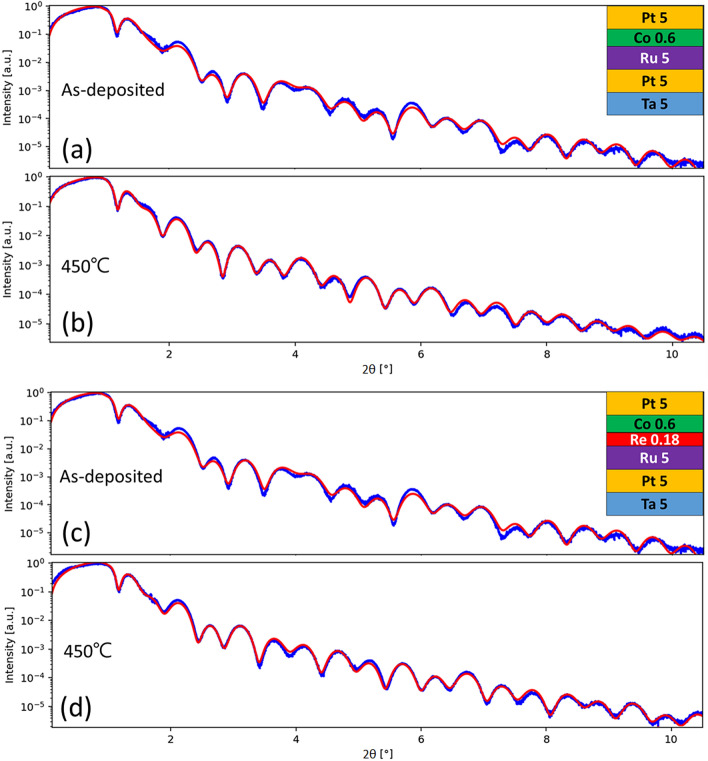
XRR spectra and fitting. (**a**-**b**) are for sample A (Ta 5/Pt 5/Ru 5/Co 0.6/Pt 5) with Ru/Co interface and (**c**-**d**) are for sample B (Ta 5/Pt 5/Ru 5/Re 0.18/Co 0.6/Pt 5) with Ru/Re/Co interface. (**a**) and (**c**) are for as-deposited samples, and (**b**) and (**d**) are for annealed samples at 450 °C for 1 h. The blue curves are experimental ones and the red curves are their corresponding fitting curves. The fitted roughness at Ru/Co is 0.39 and 0.55 nm for the as-deposited and annealed samples, respectively. The fitted roughness at Re/Co is 0.37 and 0.45 nm for the as-deposited and annealed samples, respectively.

In order to quantize the interfacial difference and the function of Re during annealing, we use X-ray reflectometry (XRR) analysis. Because of complicated multilayer structure, it is not easy to fully fit the experimental data of XRR. Therefore, we prepared samples in simplified structures to highlight the interface we would like to investigate. Three samples were grown: sub//Ta 5/Pt 5/Ru 5/Co 0.6/Pt 5 (sample A), Ta 5/Pt 5/Ru 5/Re 0.18/Co 0.6/Pt 5 (sample B), Ta 5/Pt 5/Co 0.6/Ru 5 (sample C) and Ta 5/Pt 5/Co 0.6/Re 0.18/Ru 5 (sample D). The interface we are interested in is underlined. Sample A (C) corresponds to the case for top (bottom) interface of a single Ru spacer in SAF structure. Sample B (D) corresponds to the case for upper (lower) Re insertion of a composite Ru/Re (Re/Ru) spacer in SAF structure. XRR results for the as-deposited samples and samples annealed at 450 °C, shown in Fig. 5, reveal clearly oscillating spectra. By fitting the spectra, we can get the information of interfacial roughness^19^. Since the sample structure is quite similar, after the spectra fitting, we mainly focus on the interfaces at Ru (Re) contacting the ferromagnetic layer. In sample A case, the fitted roughness at Ru/Co (top interface of Ru) is 0.39 nm for as-deposited state and 0.55 nm for annealed state, respectively, shown in Fig. 5a,b. The 0.16 nm increased roughness at the top interface can be considered as the average mixing thickness formed by annealing. For sample B, the fitted roughness of Re/Co (Re upper insertion) is 0.37 nm in the as-deposited state (Fig. 5c). Because the Re layer is quite thin so it grows conformally on Ru with similar roughness. After 450 °C annealing, Re/Co roughness becomes 0.45 nm, but Ru/Re interfacial roughness remains at 0.38 nm (Fig. 5d). The increased Re/Co roughness is only 0.08 nm, half of Ru/Co in sample A, suggesting that Re insertion on top of Ru indeed can significantly suppress the inter-diffusion. For sample C, the fitted interfacial roughness at Co/Ru (bottom interface of Ru) is 0.32 nm at as-deposited state and 0.42 nm for annealed state, shown in Fig. S3 of Supplementary Information. The increased roughness 0.10 nm at the bottom Co/Ru is much smaller than that of the top Ru/Co interface for the single Ru spacer and comparable to the case with Re upper insertion. For sample D, fitted interfacial roughness at the interface of Re/Ru, is 0.34 nm at as-deposited state and 0.43 nm for annealed state, shown in Fig. S3 of Supporting Information. The increased roughness 0.09 nm is similar to sample C, revealing that the bottom insertion of Re does not significantly suppress diffusion. The fitted interfacial roughness from XRR data consists with our observation for the J_ex_ variations with different spacer, shown in Fig. 3. The XRR data clearly reveal that the inter-diffusion at the top Ru/Co is more severe after 450 °C annealing; therefore, the upper insertion of Re (Ru/Re spacer) can effectively suppress the inter-diffusion, leading to larger J_ex_ than the one with the Ru spacer after annealing.

**Figure 6:**
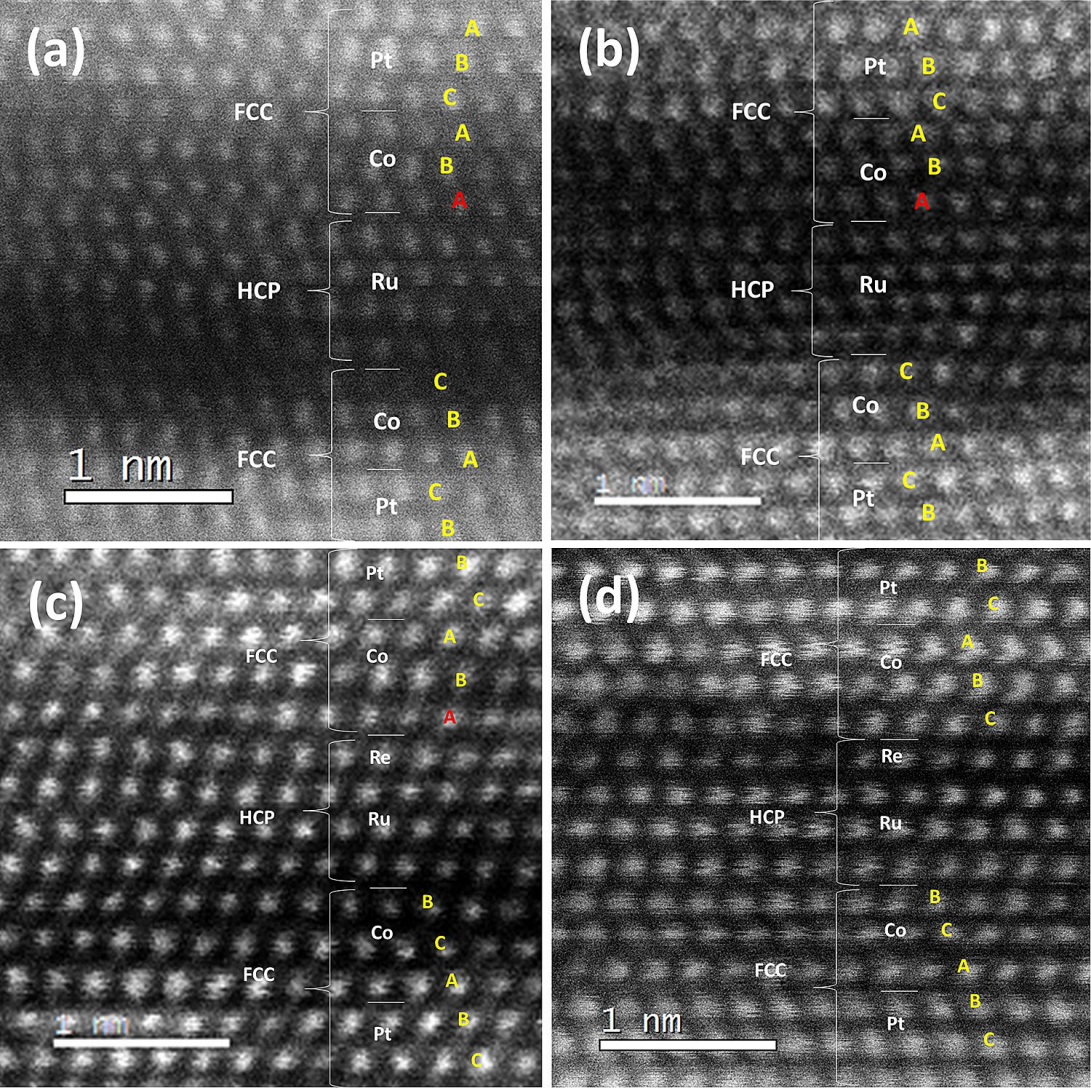
STEM-HAADF image of SAFs. (**a**) and (**b**) SAF with a single Ru spacer in the as-deposited and annealed state, respectively. (**c**) and (**d**) SAF with a Ru/Re composite spacer at as-deposited and annealed state, respectively. The elements and crystal structures are indicated. The types of atom positions are noted by A, B and C in yellow and the stacking fault layer is marked in red.

To clarify the mechanism, which results in the difference between upper and lower interfaces, we took a close look into the microstructure by using STEM. By STEM-HAADF, shown in Fig. 6, we can clearly observe the atoms and how they stacked in the layers. In typical Ru-based perpendicular SAF system, [Co/Pt] multilayers grow along FCC [111] to generate large perpendicular anisotropy due to the interfacial interaction between Co and Pt^20–22^. Therefore, a high quality Pt seed layer is usually deposited first to provide strong (111) texture, leading to highly (111)-textured [Co/Pt]_n_. The STEM-HAADF images, shown in Fig. 6, reveal that the bottom Pt/Co layers are well grown in a regular FCC order A-B-C, in which atoms are arranged in three kinds of atomic positions of the FCC structure. On top of Pt/Co, HCP Ru grows along [0001] with the close-packed plane parallel to FCC (111). The upper [Co/Pt] layers are expected to be perfectly aligned FCC (111) as well. However, for top Co/Pt, the first Co layer does not follow the regular A-B-C order of FCC, as shown in Fig. 6a, but forms a stacking fault, which is a kind of misalignment in a serial growth of lattice and may occur on the close-packed plane of FCC system. In this fault layer, atoms occupy the position of A-site instead of C-site, and form A-B-A ordering, which becomes HCP-like structure for the Co layer. It is known that Co can have two kinds of structure HCP and FCC and generally, a thin Co layer in Co/Pt prefers to form an FCC structure^23^. However, due to the existence of HCP Ru spacer in the SAF structure, Co is possible to initially grow with HCP sequence. The sample with the Ru/Re composite spacer also exhibits similar situation at the interface of Re/Co because Re is HCP as well. The fault layer stores additional energy, called stacking fault energy, compared to the well-ordered FCC structure. During annealing, the extra stored energy in the fault layer may provide additional driving force to make atoms migrate more easily. During the migration of Co atoms, some vacancies or wider atomic spacing might be temporarily induced so that Ru atoms have more chances to intermix with Co. Consequently, Ru and Co atoms may probably form substitutional diffusion so that the lattice or texture seems not to be deformed significantly but the interface of Ru/Co becomes intermixing after annealing, resulting in the degradation of RKKY strength. In addition, due to severe intermixing at Ru/Co interface, the first Co-layer atoms seem not successfully to recover their positions into FCC sequence but remains in the HCP structure instead, as shown in Fig. 6b. In contrast, when Re layer is inserted between Ru and Co, as shown in Fig. 6c, because Re is much heavy and stable than Ru, the migration is not as easy as Ru. During the annealing, the Re behaves as the diffusion barrier with much reduced migration; therefore, the intermixing is not so severe, leading to higher J_ex_ than that of the Ru spacer. Notice that the Co atoms in the fault layer recover their positions back to the right FCC sequence, as shown in Fig. 6d. Because Re/Co intermixing is significantly suppressed due to the Re characteristics, Co atoms can have a chance to move back their preferred positions. On the other hand, at the lower interface Co/Ru, the Co layer lattice is a well-aligned FCC without the formation of stacking fault. Consequently, less atomic migration in the bottom Co layer during annealing reduced the intermixing at the Co/Ru bottom interface. The presence of stacking fault may explain the different increases of interfacial roughness at the top and bottom Ru interface after annealing, obtained in XRR analyses. In addition, it also explains why the upper Re insertion (Ru/Re) is more effective than the bottom insertion (Re/Ru) to keep high J_ex_ after annealing. Our findings might also partially explain why the Ir spacer can possesses extremely high thermal durability. Ir is FCC so the whole SAF layers probably could stack from down to up in a proper FCC sequence without formation of stacking fault, as observed in the Ru case.

## Conclusion

In this work, we demonstrate an interfacial engineering by Re insertion in Ru-based SAF structure to enhance the interlayer coupling after high temperature annealing. The composite spacer Ru/Re reveals significantly improved thermal tolerance compared to the single Ru spacer. We clearly show that much more severe inter-diffusion occurs during annealing at upper Ru/Co than that at lower Co/Ru interface possibly due to the formation of stacking fault at the upper interface. By inserting a monolayer of Re on top of Ru layer (Ru/Re spacer), the inter-diffusion between spacer and Co can be substantially suppressed during annealing. On the other hand, although the lower Re insertion can also help the suppression of inter-diffusion for high temperature annealing, the thin Re layer at the bottom may slightly deteriorate the Ru texture, leading to reduced J_ex_. Therefore, the optimized spacer structure would be a bilayer composed of Ru/Re, which can effectively be against the inter-diffusion at the upper interface and remain lower Ru/Co interface to provide strong coupling strength. The interlayer coupling strength with Ru/Re spacer can remain more than half of the as-deposited value and reach 0.57 erg/cm^2^ even after 1 h annealing at 450 °C, much higher than the value of single Ru spacer. The good performance upon 450 °C makes a wider process window for MTJs to be integrated to CMOS. Although our demonstrated coupling strength is still inferior to that of the Ir spacer, the cost of Re is much more affordable. Furthermore, our findings on the working mechanism based on the microstructure analysis can serve as a guideline for the material selection and layer structure design, not only for the SAF structure but also for other layers in the p-MTJs.

## Methods

### Samples preparation

The SAF structure consists of Si/SiO_2_//Ta 5/Pt 5/[Co 0.6/Pt 1/Co 0.6]/Re t_1_/Ru t_0_/Re t_2_/[Co 0.6/Pt1/Co 0.6]/Pt 2. All the numbers for layer thickness in this work are presented in nanometers. Re was inserted at top and/or bottom of Ru as the key player for interfacial engineering. The thickness of Ru and Re are varied as t_0_, t_1_, and t_2_ for comparison. These samples were deposited on thermally oxidized Si (100) substrates by using a high vacuum DC magnetron sputtering system with base pressure of 8 × 10^−8^ torrs. After deposition, samples were annealed at varied temperatures in a vacuum furnace with pressure better than 2 × 10^−5^ torrs for 1 h.

### Characteristic methods

The magnetic properties were obtained from hysteresis loops measured by vibrating sample magnetometer (VSM, PMC, Micromag Model 3900). The crystalline information was examined by X-ray diffractometer (XRD, Shimadzu, LabX XRD-6000, Cu-Kα1). The interfacial roughness was characterized by the X-ray reflectometry (XRR, PANalytical, PANalytical-X'Pert PRO, Cu-Kα1) and fitted by the software GenX. Microstructure images were taken by scanning transmission electron microscope (TEM, JEOL, JEM-ARM200FTH, 200 kV) with spherical-aberration corrector and high angle annual dark field detector. (HAADF).

## Supplementary Information

Supplementary Information.
