# Supplementary material for: High thermal durability of Ru-based synthetic antiferromagnet by interfacial engineering with Re insertion
Source: Sci Rep. 2021 Jul 26;11:15214. doi: 10.1038/s41598-021-94640-4 (PMC8313549; doi:10.1038/s41598-021-94640-4)
Supplement: Supplementary file 1 — Supplementary Information. [file 41598_2021_94640_MOESM1_ESM.docx]

**High Thermal Durability of Ru-based Synthetic Antiferromagnet by Interfacial Engineering with Re Insertion**

Chun-Liang Yang^1^, Chih-Huang Lai^1,*^

^1^Department of Materials Science and Engineering, National Tsing Hua University

No.101, Section 2, Kuang-Fu Road, Hsinchu 30013, Taiwan

^*^chlai@mx.nthu.edu.tw

**S1. J_ex_ of spacers with different thickness**

**
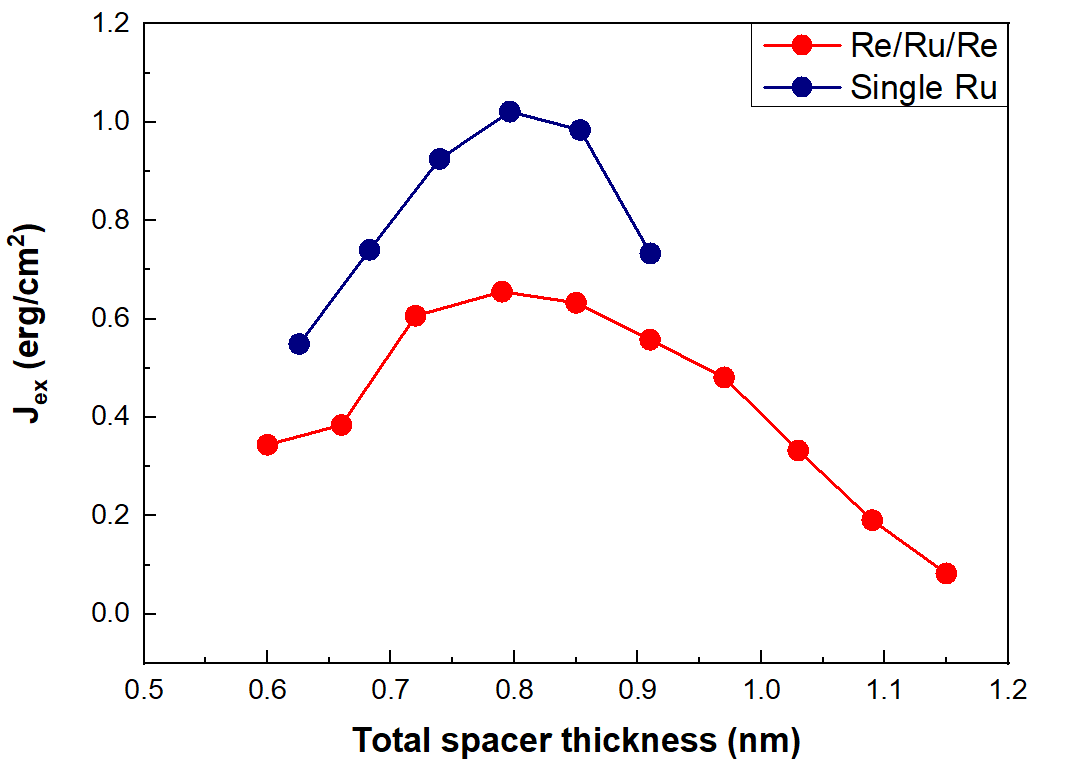
**

**Figure S1.** Effects of total spacer thickness on RKKY interlayer coupling

We prepared samples with spacer of a single Ru layer and sandwiched structure Re 0.18nm /Ru t/Re 0.18nm, respectively. J_ex_ is plotted as a function of thickness to show the dependence of J_ex_ on the total spacer thickness in the as-deposited state for the sample with a single Ru spacer (blue) and a composite spacer Re/Ru/Re (red). For the sample with total spacer thickness below 0.6 nm, Ru is too thin (<0.24nm) in the Re/Ru/Re composite spacer to provide sizable RKKY coupling. Compared to the sample with a single Ru spacer, the sample with composite spacer reveals similar dependence of interlayer coupling on spacer thickness, which has a maximum value around 0.79 nm (Ru 2^nd^ RKKY anti-parallel coupling peak).

**S2. XRD measurement**


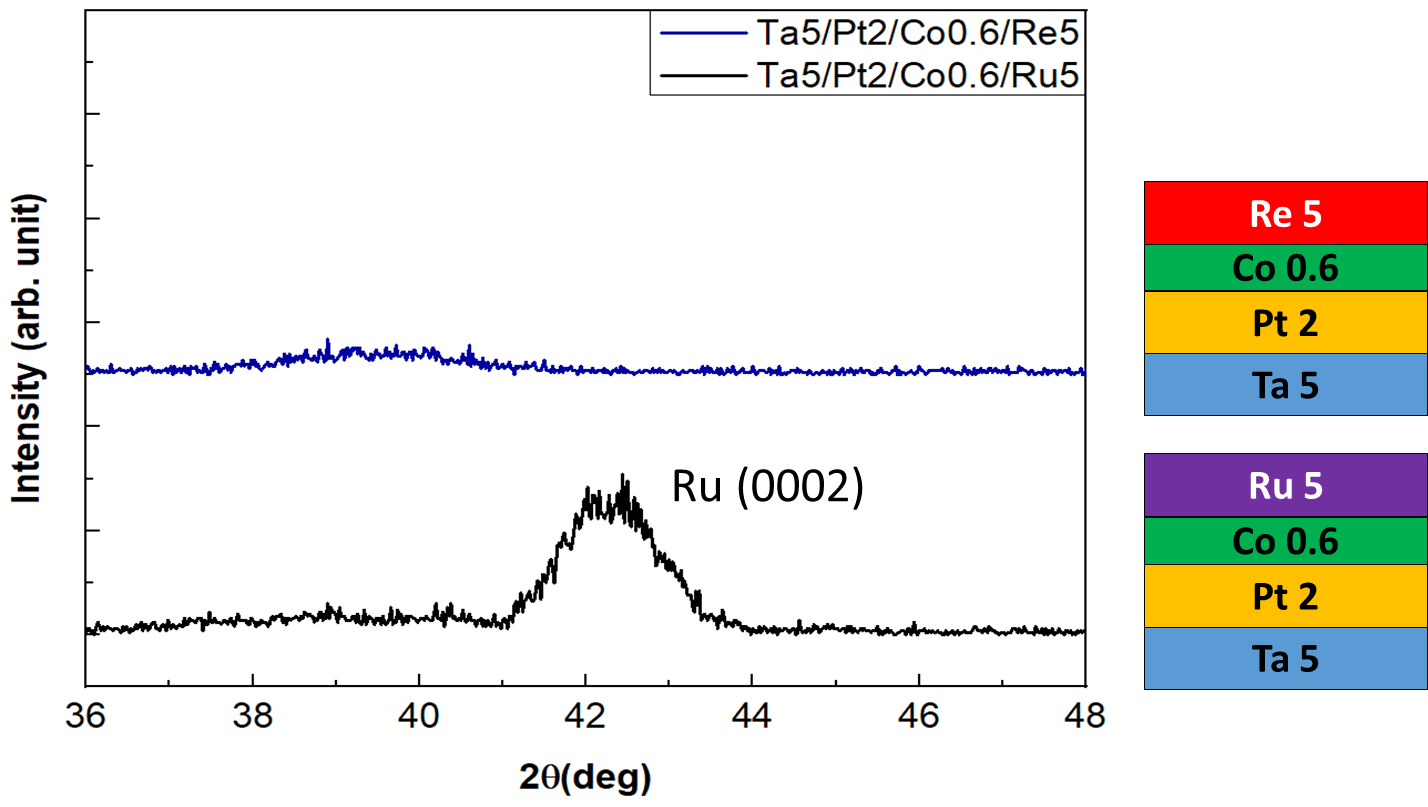


**Figure S2.** XRD spectra for Ta/Pt/Co/Ru (or Re).

We prepared samples with the same underlayers, Ta 5/Pt 2/Co 0.6 (numbers are thickness in nanometers), to verify the crystallinity of Ru and Re. Because Pt is well known to grow a strong (111) texture on Ta, we decrease its thickness to avoid the peaks overlapping. For the sample with Ru, Ru shows a clear peak of (0002) on FCC (111) Pt/Co. In contrast, Re does not build a clear textured structure like Ru.

**S3. XRR measurement**


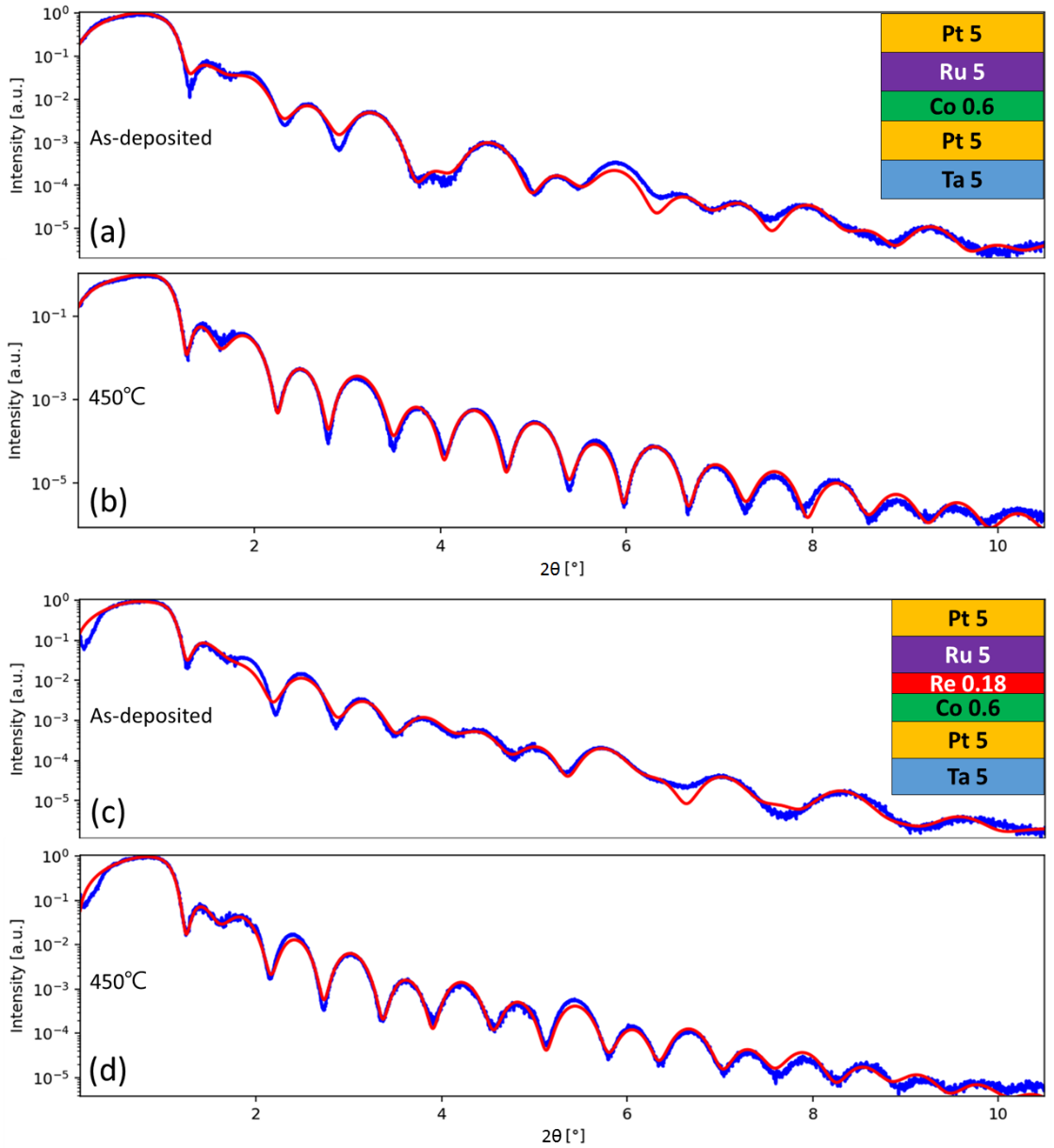


**Figure S3. XRR spectra and fitting curve.** (a) and (b) are as-deposited and annealed states of sample C, Ta 5/Pt 5/Co 0.6/Ru 5/Pt 5. (c) and (d) are as-deposited and annealed states of sample D, Ta 5/Pt 5/Co 0.6/Re 0.18/Ru 5/Pt 5

We prepared sample C, Ta 5/Pt 5/Co 0.6/Ru 5/Pt 5 and sample D, Ta 5/Pt 5/Co 0.6/Re 0.18/Ru 5/Pt 5, which corresponded to the situation of lower interface of Ru with and without Re insertion. The fitted interfacial roughness at Co/Ru are 0.32 nm and 0.42 nm before and after annealing for sample C, respectively. The interfacial roughness at Co/Re are 0.34 nm and 0.43 nm before and after annealing in sample D, respectively.
